# Unmasking the uncommon: retroperitoneal Leiomyosarcoma case report

**DOI:** 10.1093/omcr/omaf002

**Published:** 2025-03-28

**Authors:** Boujguenna Imane, Mohammed Essaid Ramraoui, Fatima Boukis, Faisal ElMouhafid

**Affiliations:** Guelmim Faculty of Medicine and Pharmacy, Ibn Zohr Agadir University, Guelmim Morocco; Guelmim Military Hospital Moulay El Hassan General Surgery Department, Guelmim 81000, Morocco; Al AMAL Pathological Anatomy Laboratory, Guelmim 81000, Morocco; Guelmim Military Hospital Moulay El Hassan General Surgery Department, Guelmim 81000, Morocco

**Keywords:** Leiomyosarcoma, retroperitoneal sarcoma, soft tissue sarcoma, immunohistochemistry, surgical resection

## Abstract

Leiomyosarcoma is a rare subtype of soft tissue sarcoma originating from smooth muscle cells. The clinical presentation varies based on the tumor’s location. We report the case of a 63-year-old woman with no significant medical history, who presented with persistent epigastric pain. A suspected lymphadenopathy was found on a CT scan. Following surgical excision, histopathology and immunohistochemistry confirmed the diagnosis of leiomyosarcoma, negative for CD117 and Dog1, but positive for H-Caldesmon. Retroperitoneal leiomyosarcoma is aggressive and rare, making diagnosis difficult prior to histopathology. Complete surgical resection with negative margins is the gold standard of treatment, though it can be challenging to achieve. A multidisciplinary approach is crucial to improve survival and quality of life. The patient is under regular follow-up and remains free of recurrence six months post-operatively.

## Introduction

Retroperitoneal tumors are uncommon, typically arising in the retroperitoneal and subperitoneal spaces. These tumors, often malignant, are diagnosed late. Sarcomas account for 10%–15% of retroperitoneal tumors [1], and leiomyosarcomas represent 5%–10% of all soft tissue sarcomas [2]. About half of leiomyosarcomas occur in the retroperitoneal or abdominal region, with the uterus being the most common site [3]. Diagnostic imaging, particularly CT, is essential in identifying these tumors, although histopathology remains the definitive method for diagnosis. This case highlights the diagnostic challenges presented by retroperitoneal leiomyosarcoma, initially suspected to be lymphadenopathy.

## Case report

**Figure 1:**
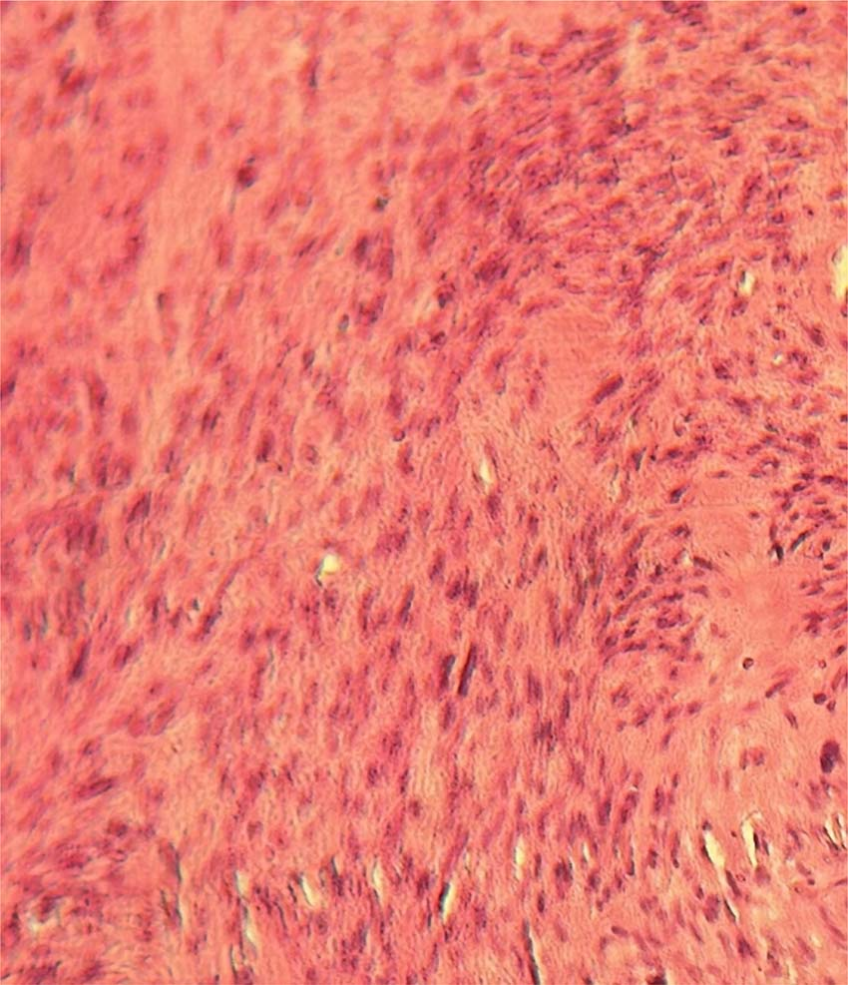
Tumor with moderate cellular density (×25).

**Figure 2:**
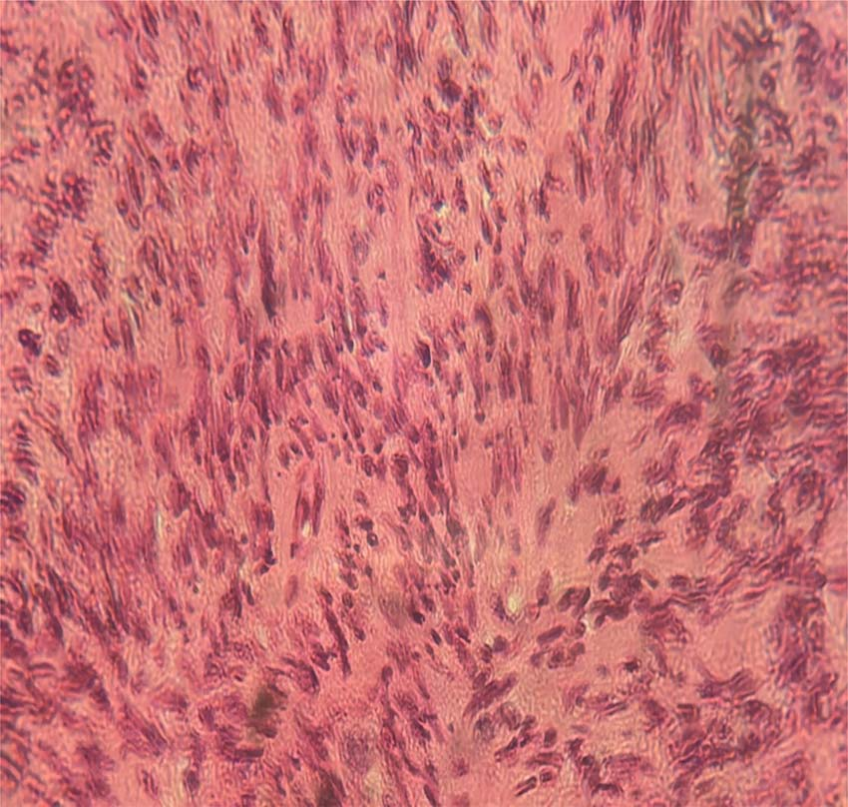
Tumor cells were spindle-shaped, occasionally large, with elongated, hyperchromatic nuclei, sometimes vesicular, and irregular contours (×40).

**Figure 3:**
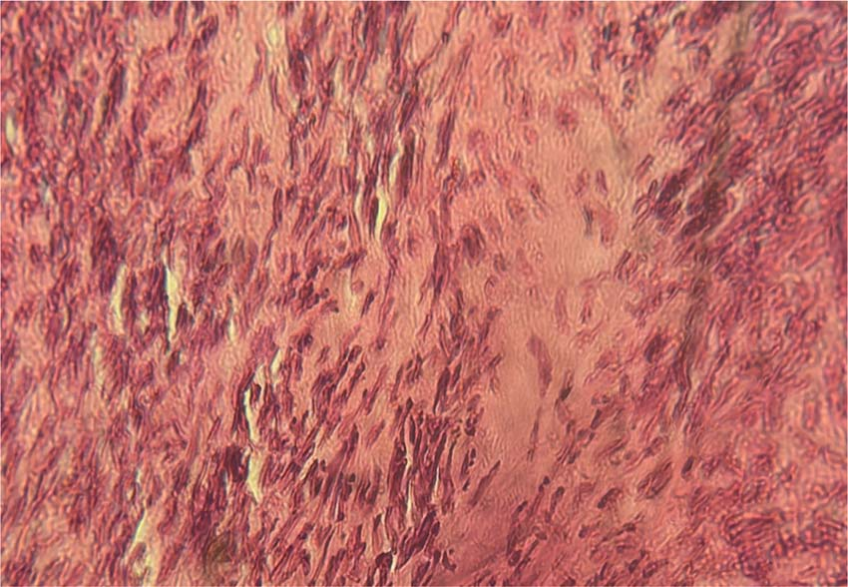
Tumor with microfoci of tumor necrosis (×40).

**Figure 4:**
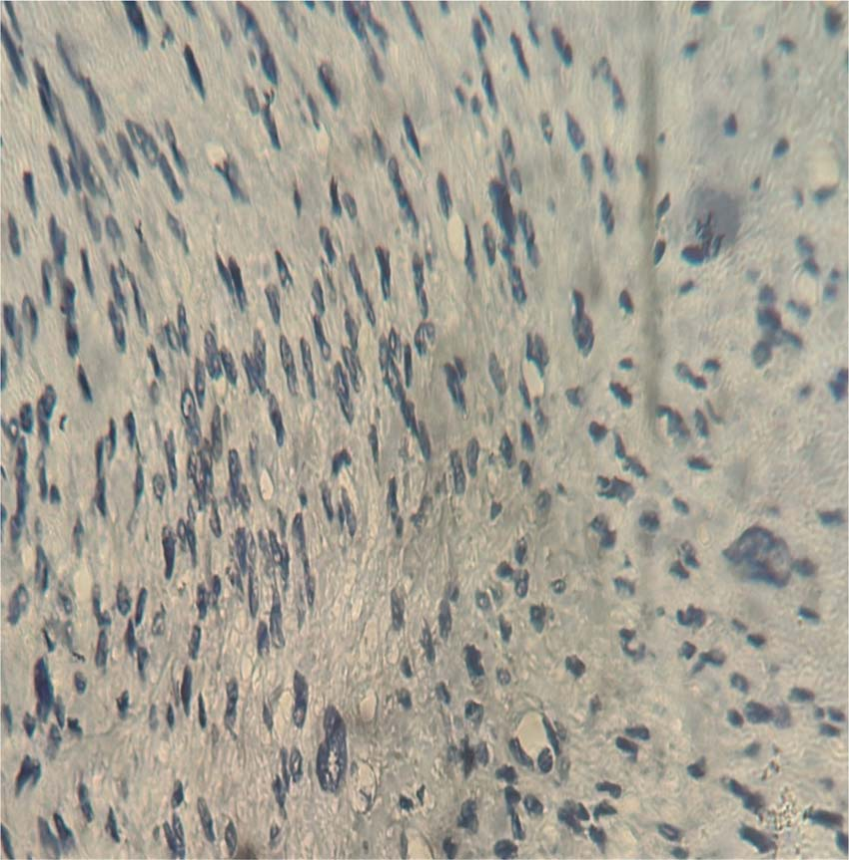
Negative staining CD117 (×40).

**Figure 5:**
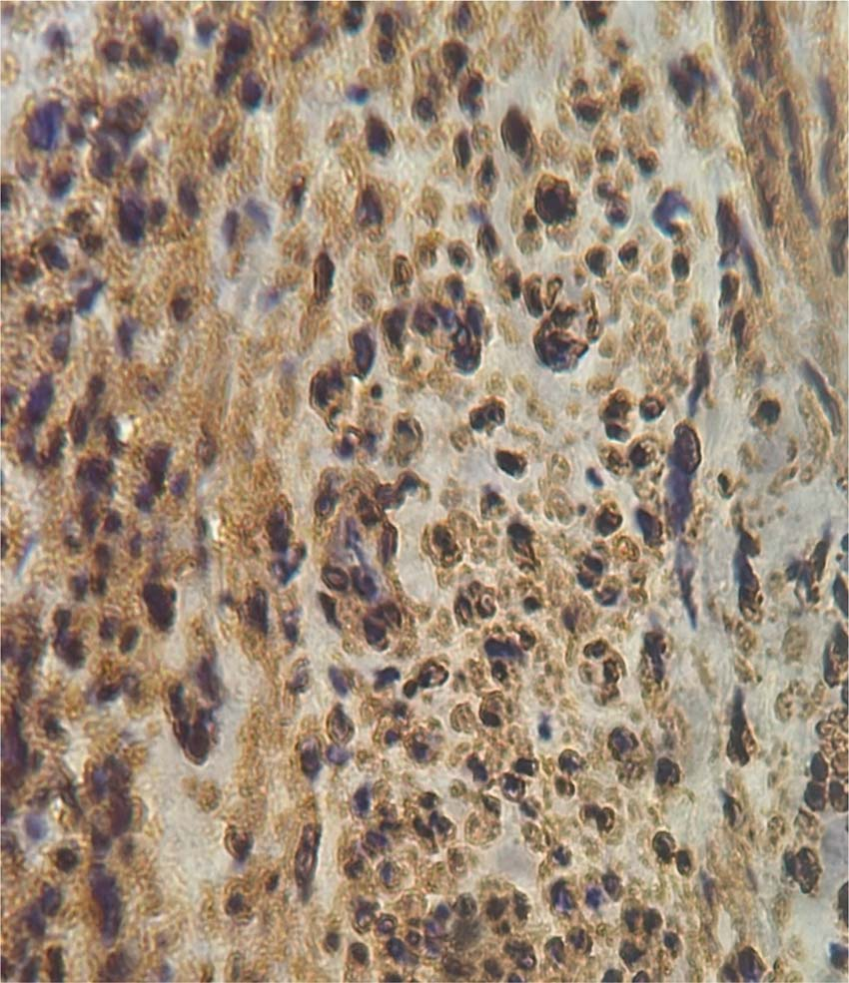
Positive staining alpha smooth muscle actin (×40).

**Figure 6:**
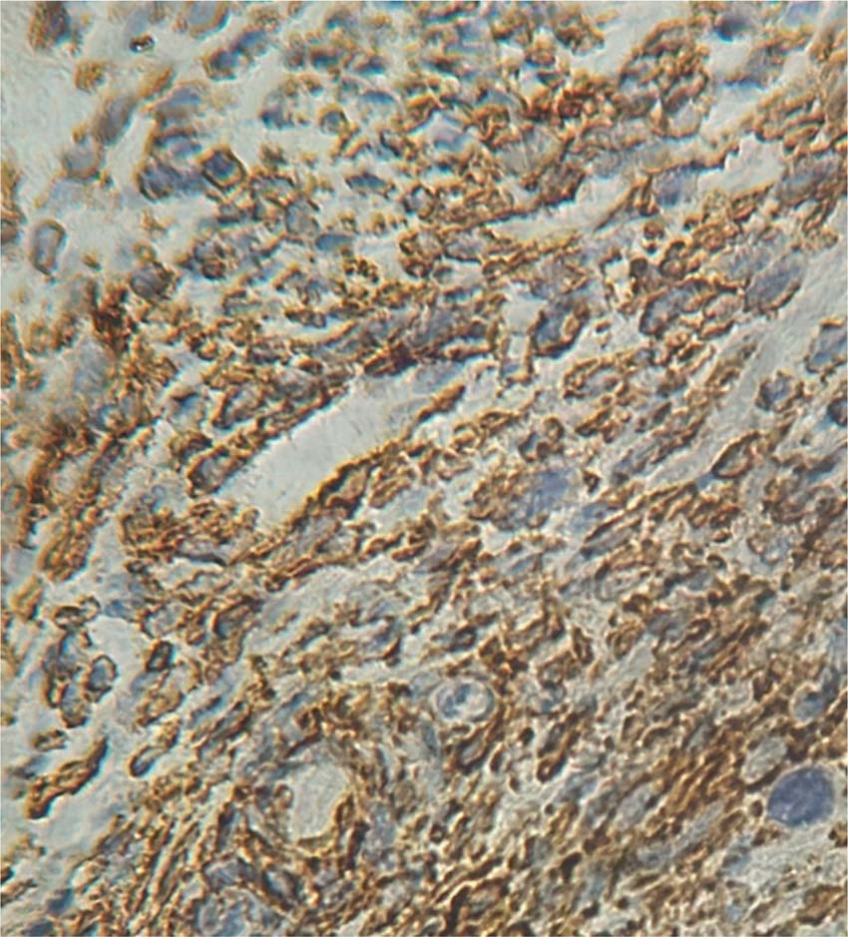
Positive staining H-Caldesmon (×40).

A 63-year-old woman, with no significant medical history, presented with chronic epigastric pain. After several inconclusive tests, including endoscopy, repeated abdominal ultrasounds detected a suspicious mass. A CT scan identified a well-defined, lobulated 34 × 24 mm mass in the omental bursa, initially suggestive of lymphadenopathy. The mass was surgically excised. Histopathological examination revealed a spindle-cell tumor with moderate cellularity, elongated hyperchromatic nuclei, and a mitotic index of 5 per 10 high-power fields. The stroma was fibrous, with minimal inflammatory infiltrate (Figs 1–3). Immunohistochemistry confirmed leiomyosarcoma, with negative CD117 (Fig. 4) and Dog1, and positive Alpha Smooth Muscle Actin (Fig. 5) and H-Caldesmon staining (Fig. 6). The tumor was classified as grade 1 (FNCLCC classification). Given the negative surgical margins, no adjuvant treatment was recommended, and the patient remains under clinical follow-up, with no recurrence observed at six months.

## Discussion

Retroperitoneal sarcomas are rare, accounting for 10%–15% of all soft tissue sarcomas [1]. These tumors are typically large and advanced at diagnosis due to their insidious presentation. The median size at detection is approximately 15 cm [2], although in our case, the tumor was significantly smaller, measuring only 34 mm. Retroperitoneal leiomyosarcomas frequently cause compressive symptoms, such as abdominal pain, depending on the size and location of the tumor. In some instances, patients with rapidly growing high-grade tumors may present with systemic symptoms, such as weight loss or fatigue [3, 4]. Retroperitoneal leiomyosarcomas can present with an intraluminal component in approximately 38% of cases [4], which may involve critical structures such as the inferior vena cava (IVC). Tumors involving the IVC can obstruct hepatic veins, causing Budd-Chiari syndrome with symptoms such as hepatomegaly, jaundice, and ascites. If the middle or lower IVC is affected, patients may experience renal dysfunction or lower extremity edema, respectively. In our case, there was no evidence of IVC involvement, which likely contributed to the localized symptoms of epigastric pain. Diagnosis is primarily guided by imaging, with CT scans being the preferred method for assessing tumor size, location, and involvement of adjacent structures. Although MRI can provide additional soft tissue characterization, in our case, CT was the key modality used. It is important to note that leiomyosarcomas lack specific imaging features that distinguish them from other soft tissue tumors, such as gastrointestinal stromal tumors (GIST) or lymph node lesions, which can complicate preoperative diagnosis. In our patient, the initial CT findings suggested lymphadenopathy. Only after histopathological examination and immunohistochemical analysis, which ruled out GIST (with CD117 and Dog1 negativity), was the diagnosis of leiomyosarcoma confirmed with positive H-Caldesmon staining. PET scans can play a valuable role in detecting recurrences, particularly post-surgery. Approximately 90% of recurrences can be identified through PET, which can guide future diagnostic and therapeutic decisions [5, 6]. Histopathological examination remains the gold standard for diagnosing retroperitoneal leiomyosarcoma. The main histological features include cytological atypia, tumor necrosis, and mitotic activity (>10 mitoses per 10 high-power fields) [2]. The immunohistochemical profile is essential for differentiating leiomyosarcomas from other mesenchymal neoplasms, particularly GIST, as seen in our case, where CD117 and Dog1 were negative, while H-Caldesmon and Alpha Smooth Muscle Actin were positive. These markers confirm the smooth muscle origin of the tumor and exclude other differential diagnoses, such as benign leiomyoma or smooth muscle tumors of uncertain malignant potential [7, 8]. The treatment of choice for leiomyosarcoma is complete surgical resection with negative microscopic margins, although achieving these margins can be challenging, particularly in the retroperitoneal space. Extended resections may be necessary to avoid tumor spillage and ensure clean margins, often involving adjacent organs. In our case, complete resection was successfully achieved, and no adjuvant therapy was recommended based on the multidisciplinary team’s evaluation. However, close follow-up is essential due to the high risk of recurrence [9, 10]. Retroperitoneal leiomyosarcoma is an aggressive and rare tumor, and definitive diagnosis before histopathological evaluation remains difficult. Nevertheless, surgical excision with negative margins offers the best chance for long-term survival. A multidisciplinary approach is crucial to optimizing both survival and quality of life in patients with retroperitoneal leiomyosarcoma.
